# 1815. Clinical Characteristics of Infections due to Staphylococcus Lugdunensis Compared to Other Staphylococcal Infections

**DOI:** 10.1093/ofid/ofad500.1644

**Published:** 2023-11-27

**Authors:** Souad Bou Harb, Youssef Zougheib, Ayah Matar, Reem Mansour, Zeina Kanafani

**Affiliations:** American University of Beirut, Beirut, Beyrouth, Lebanon; American University of Beirut, Beirut, Beyrouth, Lebanon; American University of Beirut, Beirut, Beyrouth, Lebanon; American University of Beirut, Beirut, Beyrouth, Lebanon; American University of Beirut Medical Center, Beirut, Beyrouth, Lebanon

## Abstract

**Background:**

*Staphylococcus lugdunensis* (SL) belongs to the group of coagulase negative Staphylococci (CoNS). SL infections may have a more virulent course compared to other CoNS, bearing resemblance to infections caused by *S. aureus* (SA). This study aims to investigate the clinical characteristics of SL infections at a tertiary care center in Lebanon.

**Methods:**

We enrolled all patients presenting from 2017 to 2021 with positive cultures for SL from four clinical sites (urine, respiratory, wound, and blood). We then selected controls with SA matched 1:1 by site of infection. Since CoNS is not a well-described respiratory pathogen, CoNS were only matched to urine, wound, and blood. Patients who were deemed to have colonization were excluded. We determined the risk factors and outcomes associated with SL infections compared to SA and CoNS infections. We then identified predictors of mortality in the SL cohort.

**Results:**

We identified 101 patients with SL infection, who were matched to 103 patients with SA and 97 patients with CoNS. Most SL isolates were obtained from wound samples (72%), followed by urine (16%), blood (8%) and sputum (4%). Compared to SA, SL patients were less likely to be admitted to the hospital for more than 6 days within the past 30 days (8% vs. 31%; p< 0.01). On the other hand, compared to CoNS, patients in the SL cohort were more likely to have a history of endocarditis (6% vs. 0%; p=0.01). There were no other significant differences between baseline characteristics of SL, SA, and CoNS patients. As far as outcomes, compared to the SA group, patients with SL were more likely to develop renal insufficiency (odds ratio [OR] 4.9; 95% confidence interval [CI] 1.0-23.5) and less likely to have persistent bacteremia (OR 0.5; 95% CI 0.4-0.6). No difference in outcomes was observed between the SL and CoNS cohorts. The mortality rate among SL patients was 8%. The predictors of mortality in this cohort are found in the table.

Bivariable and multivariable analysis of predictors of mortality among patients with SL infections

**Figure:**
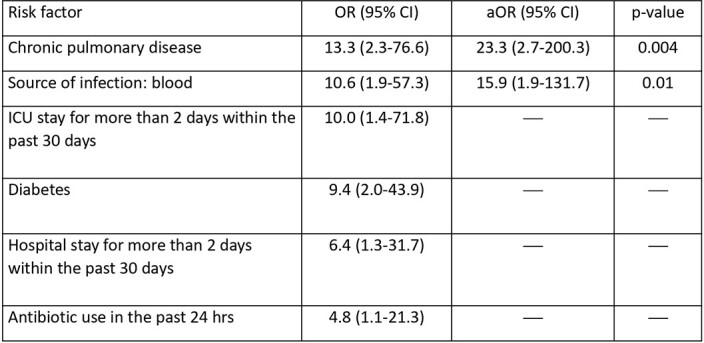

**Conclusion:**

SL appears to be more associated with patients in the community rather than the hospital setting and may be more likely to occur in patients with damaged heart valves. Chronic pulmonary disease and a blood source of infection are associated with a fatal outcome in SL patients.

**Disclosures:**

**All Authors**: No reported disclosures

